# Dual trigger and the impact on oocyte quality and embryo development: a Brazilian cohort

**DOI:** 10.5935/1518-0557.20230048

**Published:** 2023

**Authors:** Isadora Ferreira Kozlowski, Vinicius Bonato da Rosa, Camila Dutra de Souza Francisquini, Alessandro Schuffner

**Affiliations:** 1 Pontifícia Universidade Católica do Paraná, Curitiba, Paraná, Brazil; 2 Brown Fertility - Florida Fertility Clinics, Jacksonville, Florida, United States; 3 Conceber Centro de Medicina Reprodutiva, Curitiba, Paraná, Brazil. Zip Code: 80240-210

**Keywords:** ovarian stimulation, pituitary hormones, oocyte triggering, *in vitro* fertilization, oocyte maturation

## Abstract

**Objective:**

We aimed to analyze controlled ovarian stimulation using GnRH antagonist in association with hCG (dual triggering) versus hCG alone (conventional triggering) for final oocyte maturation triggering in a population of unselected Brazilian women.

**Methods:**

This was a retrospective observational study of IVF medical records between January 2019 and March 2020. Data from 335 women with infertility were included for study. All patients were divided into hCG trigger (control group; n=178) and dual trigger (n=157).

**Results:**

The number of total oocytes and the number of inseminated oocytes were all significantly higher with the dual trigger protocol compared to hCG-only trigger. However, there is no significant difference in patient age, type of infertility, number of *in vitro* matured oocytes, trigger day, endometrial thickness, AMH concentration, the number of follicles, the number of mature oocytes and the number of fertilized oocytes.

**Conclusions:**

Using the dual trigger protocol improved the number of total oocytes retrieved and the number of inseminated oocytes.

## INTRODUCTION

One of the major factors influencing the ability to conceive is the female age (Datta *et al*., 2016; Attali & Yogev, 2021) Woman fertility starts to decline around 25-30 years of age and the average age at last birth is 40-41 years in most studied populations experiencing natural conception (Vander Borght & Wyns, 2018; Ahmed *et al*., 2020; Irani *et al*., 2020). Assisted reproductive technologies (ART) is often the primary choice to treat human infertility (de Ziegler *et al*., 2019). In normal menstrual cycle, spontaneous ovulation is preceded by a surge in both follicle stimulating hormone (FSH) and luteinizing hormone (LH) to induce final oocyte maturation. However, in the conventional controlled ovarian stimulation (COS) regimen, it is not possible to use LH as ovulation triggering since it has a short half-life in plasma (< 60 minutes) (Casper, 2015; Haas *et al*., 2020; Tan *et al*., 2020; Hu *et al*., 2021). Therefore, in COS, final follicular maturation is triggered by human chorionic gonadotropin (hCG) which acts as a surrogate to the naturally occurring LH surge (Ding *et al*., 2017; Zhang *et al*., 2017; Ali *et al*., 2020; Ben-Haroush *et al*., 2020; Haas *et al*., 2020; Albeitawi *et al*., 2022; Yan *et al*., 2022), allowing the process of final oocyte maturation, and subsequent implantation with appropriate luteal phase support in fresh transfer cycles (Tan *et al*., 2020). However, the COS protocol itself, its duration, the type, and drugs dosage are clinician-dependent factors that might affect oocyte and embryo quality (Gurbuz *et al*., 2016).

Triggering of final follicular maturation has become the subject of research interest in recent years, looking for *in vitro* fertilization (IVF) success rates improvement (Hu *et al*., 2021). Human Chorionic Gonadotropin is routinely used for inducing LH surge and consequently final oocyte maturation (Oliveira *et al*., 2016). However, triggering with hCG can have as a serious side effect the occurrence of Ovarian Hyperstimulation Syndrome (OHSS), secondary to prolonged stimulatory effects on the corpus luteum (Ding *et al*., 2017; Albeitawi *et al*., 2022).

The OHSS related to hCG triggering may be due to its half-life that is much longer than LH. Also, hCG do not have FSH activity that is an important issue for *in vitro* maturation of oocytes. The risk of OHSS can be reduced when replacing hCG by a GnRH agonist (GnRHa) (Lin *et al*., 2013; 2019; Zilberberg *et al*., 2015). The short half-life of pituitary LH (34 hours) associated with agonist-induced desensitization causes rapid and reversible luteolysis, avoiding the risk of OHSS (Martinez *et al*., 2013; Casper, 2015; Lin *et al*., 2019; Lan *et al*., 2020). Moreover, some studies have suggested that hCG triggering alone can have a negative impact on endometrial receptivity and embryo quality (Forman *et al*., 1988; Simón *et al*., 1995; 1998; Valbuena *et al*., 2001; Tavaniotou *et al*., 2002; Youssef *et al*., 2014).

In COS, GnRHa can be used as an effective and save alternative to hCG to release both FSH and LH that are critical for final oocyte maturation (Zhang *et al*., 2017; Zhang *et al*., 2021; Eser *et al*., 2018; Haas *et al*., 2020; Lan *et al*., 2020; Zhu *et al*., 2021; Albeitawi *et al*., 2022; Halim & Lubis, 2022).

Physiologically, hCG and GnRHa triggers are different. Unlike hCG triggering of late oocyte maturation, GnRHa triggering is a more physiological approach, the pituitary remains responsive to the GnRHa, similarly to what happens in natural cycles (Albeitawi *et al*., 2022; Halim & Lubis, 2022). Latterly, GnRHa have been used to inhibit premature release of LH. However, it has been preferred to use the GnRHa protocol for pituitary desensitization, as it is a more patient-friendly approach and reduces the risk of OHSS (Engmann *et al*., 2008; Ding *et al*., 2017; Eser *et al*., 2018; Halim & Lubis, 2022).

The reduced risk of OHSS has been demonstrated for high or hyper-responders when GnRHa trigger is administrated alone or in association with hCG (Engmann *et al*., 2008; Shapiro *et al*., 2011; Griffin *et al*., 2012; Pabuccu *et al*., 2015). Another advantage of these protocols is that they allow the use of a GnRHa to trigger the final maturation of the oocyte. Yet, using GnRHa as a trigger may result in improved oocyte (Humaidan *et al*., 2009) and endometrial quality (Forman *et al*., 1998; Simón *et al*., 1998) when compared to hCG regimen, since it allows a more physiological LH and FSH surge (Ding *et al*., 2017). Still, one disadvantage of the GnRHa trigger is that it cannot be used in the luteal long protocol that is already suppressed with GnRHa (Hong *et al*., 2022).

Despite proving to be an efficient drug, some problems emerged with the substitution of hCG by GnRHa as trigger. Kummer *et al*. (2013) found out triggering ovulation with GnRHa alone increased the risk of empty follicle syndrome due to a suboptimal LH surge. Multiple studies have shown that the use of a GnRHa as a trigger of final oocyte maturation in fresh cycles decreases the live births and ongoing pregnancy rates and increases early miscarriage rate. However, GnRHa is associated with the recovery of the most mature oocyte (Youssef *et al*., 2010; 2014).

This can be explained by the presence of FSH and LH peaks, since FSH induces the formation of LH receptors in granulosa luteinizing cells, oocyte nuclear maturation and cumulus expansion (Ali *et al*., 2020). Also, these poor outcomes could be owing to a luteal-phase deficiency as a result of the shorter duration and smaller amplitude of LH and FSH surge induced by GnRHa (Engmann *et al*., 2008; Haas *et al*., 2016; Ding *et al*., 2017). Yet, after appropriate luteal phase support, clinical pregnancy outcomes appeared to be similar in fresh transfer cycles (Humaidan *et al*., 2010). As such, the idea of a dual trigger was developed.

Some studies have shown that the dual (or double) trigger improves oocyte maturation while providing more sustained support for the corpus luteum (Lin *et al*., 2013; Decleer *et al*., 2014; Eftekhar *et al*., 2017). The benefits of dual trigger was reported not only in normal responders, but also in patients with diminished ovarian reserve (DOR) and poor ovarian response (POR), women with many immature oocytes in the previous cycle and in patients with suboptimal responses to trigger using only GnRHa or recombinant hCG (rhCG) in the previous cycle (Castillo *et al*., 2014; Griffin *et al*., 2014; Zilberberg *et al*., 2015; Lu *et al*., 2016; Lin *et al*., 2019; Ali *et al*., 2020; Ben-Haroush *et al*., 2020; Zhang *et al*., 2021).

It is believed that triggering with a bolus of GnRHa would reduce the risk of OHSS, while adding a reduced or standard dosage of hCG would also preserve adequate luteal function (Lin *et al*., 2019). Moreover, it has been suggested that the dual trigger approach can improve oocyte maturation, blastulation and pregnancy rates (Shapiro *et al*., 2011; Lin *et al*., 2013; Kim *et al*., 2014; Orvieto, 2015). Furthermore, the use of dual triggers which required lower dose of hCG, is more applicable in women with risk factors for OHSS (Shapiro *et al*., 2008; Humaidan *et al*., 2009; Hu *et al*., 2021).

Recently two systematic reviews (Ding *et al*., 2017; Chen *et al*., 2018) showed that although dual triggering seemed to be more favorable at improving pregnancy rates, it was equivalent to the hCG triggering in terms of the number of oocytes and mature oocytes retrieved. Because mature oocytes are a prerequisite in IVF cycles, further studies are required to better elucidate the most effective protocol. Also, for POR, the dual trigger is less clear cut (Chern *et al*., 2020). Another two randomized controlled trials comparing live birth rate after dual trigger and single hCG trigger reported conflicting results (Ali *et al*., 2020; Haas *et al*., 2020). According to O’Neill *et al*. (2016) using low-dose hCG with GnRH trigger could be a potential risk to OHSS. Despite all the progress achieved so far, the assisted reproduction outcome using one or dual trigger still remains a topic of ongoing debate. Prompted by the aforementioned observations, the aim of this study was to analyze COS using GnRHa in association with hCG (dual triggering) versus hCG alone (conventional triggering) for final oocyte maturation triggering in GnRH antagonist cycles in a population of unselected Brazilian women.

## MATERIAL AND METHOD

### Study design and participants

This is a retrospective observational study of IVF medical records between January 2019 and March 2020. The study was conducted at the “Conceber” (reproductive medical center), in Curitiba - PR, Brazil. Patients who fulfilled the criteria (women ranging from 28 to 47 years of age, undergoing IVF cycle in Curitiba - PR, Brazil) were included in this study. Exclusion criteria were as follows: (I) patients who interrupted the IVF cycle before oocyte retrieval; (II) patients that did not need GnRHa or hCG to stimulate oocyte maturation; and (III) patients with incomplete data. Following application of the exclusion and inclusion criteria, a total of 335 women with fertility problems were included for study. All patients were then divided into hCG trigger (control group; n=178) and dual trigger (n=157). The choice of hCG alone or dual trigger depended on the physician.

Endometrial thickness, AMH concentration, follicle number, number of retrieved oocytes, number of mature (MII) oocytes, number of inseminated oocytes and number of fertilized oocytes were considered as dependent variables. The patient age, infertility type (female, male or unexplained), the trigger date and number of maturated *in vitro* (MIV) oocytes were considered as independent variables.

### Treatment protocol

Recombinant FSH (Gonal f, Merck or Puregon, MSD) or HP-hMG (Menopur, Ferring) was used for COH, and flexible GnRH antagonist (Cetrotide, Merck or Orgalutran, MSD) protocol was used for pituitary suppression. Final oocyte maturation was triggered either by standard recombinant hCG (Ovitrelle, Merck) or the dual trigger: the co-administration of hCG and GnRH agonist (Decapeptyl 0.2 mg, Ferring) when at least two leading follicles measured 17 mm or more. Oocyte retrieval was performed 36-38 h following triggering. Oocytes were enzymatically denuded of cumulus cells, and the mature oocytes were inseminated by ICSI. Injected oocytes were incubated individually in pre-equilibrated culture medium in EmbryoSlideVR culture dishes (Vitrolife, Goteborg Sweden) covered with mineral oil, in an atmosphere of 5.0% O_2_ and 8.0% CO_2_.

### Statistical analysis

The data were analyzed using the MedCalc v20.006. The data normality was affeered based on the Shapiro-Wilk normality test. For ordinal variables, the Mann-Whitney test was used. Mean values were not normally distributed, so the Kruskal-Wallis test was used, with Dunn as a post-test. A *p* value of <.05 was considered statistically significant.

## RESULTS

**Figure 1:**
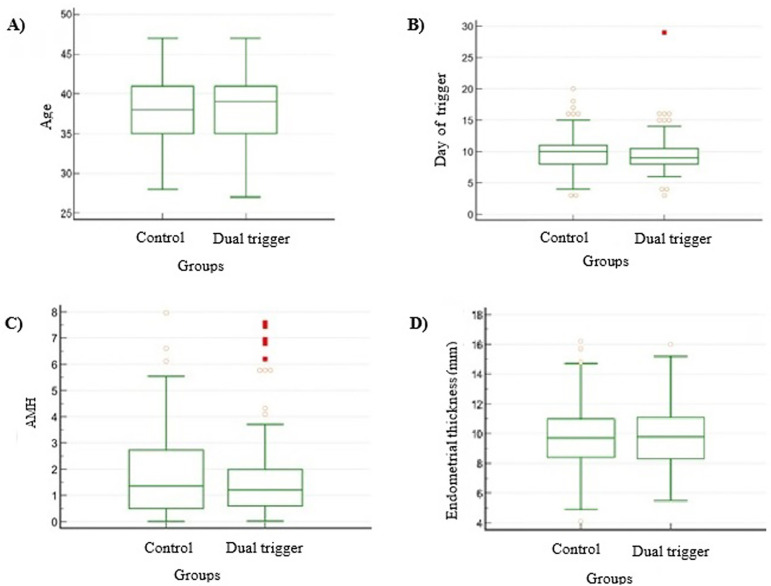
Comparison between the groups for the variables: age, day of trigger, AMH and endometrial thickness. A. Patients age (mean ± standard deviation) from control group and dual trigger group. p=0.304866; B. Trigger day (mean±standard deviation) from patients included in control group or dual trigger group. p=0.536764; C. Endometrial thickness (mean±standard deviation) from patients included in control group or dual trigger group. *p*=0.703623; D. Anti-Müllerian hormone (AMH; mean±standard deviation) from patients included in control group or dual trigger group. *p*=0.376968.

**Figure 2:**
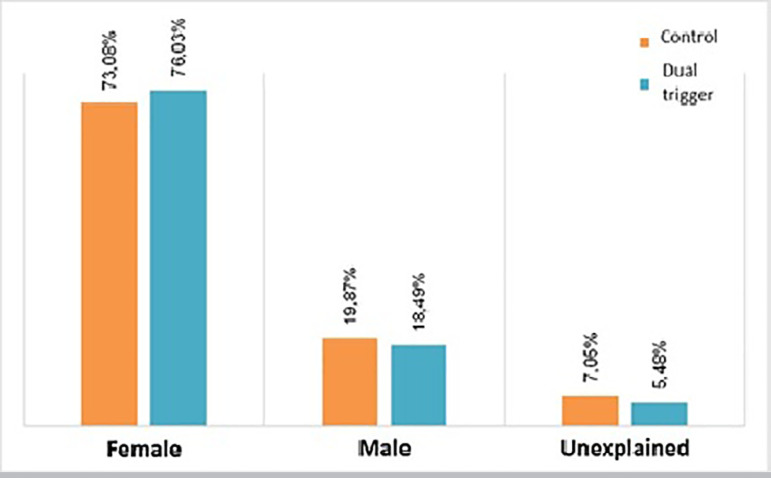
Cause of infertility (mean ± standard deviation) from control group and dual trigger group. *p*=0.9965.

Three hundred and thirty-five patients were recruited in the study. These include 178 patients in the control group (hCG trigger) and 157 patients in the dual trigger group. No differences were observed between control and dual trigger groups regarding patients age (Mean±SD: 37.7±4.1723 and 38.0±4.2787, respectively; *p*=0.304866; Figure 1A), cause of infertility (*p*=0.9965; Figure 2), day of trigger (Mean±SD: 9.8249±2.4327 and 9.6731±2.5782, respectively; *p*=0.536764; Figure 1B) and number of MIV (*p*=0.8194; Table 1).

**Table 1:** Percentage of maturated in vitro (MIV) oocytes from control group and dual trigger group. *p*=0.536764.

	Number of MIV oocytes					
	0	1	2	3	4	5
Control	91.01%	3.93%	0.00%	2.81%	1.12%	1.12%
Dual trigger	91.72%	3.18%	1.27%	1.27%	1.91%	0.64%

**Figure 3:**
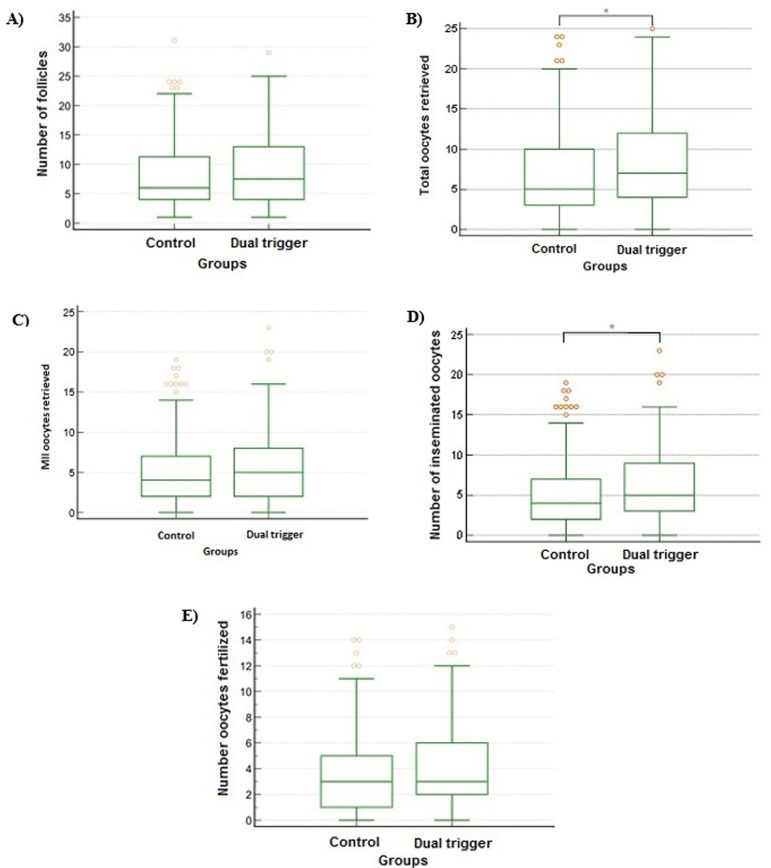
Comparison between the groups for the IFV outcomes: number of follicles, total oocytes retrieved, matured oocytes retrieved; total oocytes inseminated and total oocytes fertilized. A. Number of follicles (mean ± standard deviation) from patients included in control group or dual trigger group. *p*=0.121386; B. Total oocytes retrieved (mean ± standard deviation) from patients included in control group or dual trigger group. *p*=0.023232. (*) statistically difference. C. Matured (MII) oocytes retrieved (mean ± standard deviation) from patients included in control group or dual trigger group. *p*=0.106881; D. Total oocytes inseminated (mean±standard deviation) from patients included in control group or dual trigger group. *p*=0.049019. (*) statistically difference. E. Total oocytes fertilized (mean±standard deviation) from patients included in control group or dual trigger group. *p*=0.150925.

Regardless of trigger type, endometrial thickness (Figure 1C) was not significantly different between groups (*p*=0.703623). In the same way, AMH concentration (Figure 1D; *p*=0.376968) and number of ovarian follicle (Figure 3A; *p*=0.121386) were not different between groups.

On the other hand, the total number of retrieved oocytes (MI or MII; Figure 3B) was higher in patients treated with dual trigger (*p*=0.023232). Although, when considering only matured oocytes (Figure 3C), there was no statistically difference between groups (*p*=0.106881).

The number of inseminated oocytes (Figure 3D) was higher in patients treated with dual trigger when comparing with control group (*p*=0.049019). However, the number of fertilized oocytes (Figure 3E) did not differ between groups, even though the number of inseminated oocytes was higher in patients treated with dual trigger (*p*=0.150925).

## DISCUSSION

During IVF cycles, oocyte maturation is usually triggered by hCG for its known effect in mimicking LH surge; however, with the widespread use of the antagonist protocol, GnRHa is being more commonly employed as a trigger in order to mitigate the risk of OHSS (Albeitawi *et al*., 2022). Many studies proved its efficacy in inducing oocyte maturation (Haas *et al*., 2016; Oliveira *et al*., 2016; Elias *et al*., 2017; Zhang *et al*., 2017; Ali *et al*., 2020; Ben-Haroush *et al*., 2020; Kim *et al*., 2020), number of retrieved oocytes (Haas *et al*., 2016; Oliveira *et al*., 2016; Fabris *et al*., 2017; Zhang *et al*., 2017), fertilized oocytes (Oliveira *et al*., 2016; Elias *et al*., 2017; Lin *et al*., 2019; Albeitawi *et al*., 2022), implantation rate (Oliveira *et al*., 2016), clinical pregnancy rates (Elias *et al*., 2017; Fabris *et al*., 2017; Lin *et al*., 2019; Chern *et al*., 2020) and live birth rates (Oliveira *et al*., 2016; Elias *et al*., 2017; Lin *et al*., 2019; Chern *et al*., 2020; Hu *et al*., 2021). On the other hand, several studies have shown that dual trigger of oocyte final maturation did not improve or it was equivalent to conventional hCG triggering in terms of IVF outcome (Ding *et al*., 2017; Eser *et al*., 2018; Oliveira *et al*., 2021). Since there is conflicting evidence regarding de benefits of dual triggering protocol in human reproduction assisted techniques, we aimed to contribute with new data about the subject.

The present study showed that patient age, cause of infertility, the trigger day, endometrial thickness and AMH concentration did not differ between control and dual-trigger groups. Thus, both groups were homogeneous for those variables. According to the results from this study, dual triggering with GnRHa and hCG can be an effective alternative to hCG trigger alone, as it results in better assisted reproductive technology (ART) outcomes.

The dual trigger group showed a statistically significant higher number of total oocytes retrieved and number of inseminated oocytes compared with the control group who received standard hCG trigger alone. Those findings are in accordance to some previous studies. As expected, other studies showed that normal responders had a statistically significant higher number of total oocytes retrieved, MII oocytes and number of fertilized oocytes compared to patients who received only hCG trigger (Lin *et al*., 2013; Decleer *et al.*, 2014; Albeitawi *et al*., 2022).

Furthermore, Fabris *et al*. (2017) conducted a retrospectively study with 81 patients who had a previously IVF cycle with a high rate of immature oocytes even after hCG trigger. They found out an increase in the number of mature oocytes retrieved when using dual trigger (Fabris *et al*., 2017). Similar results were also found by other studies (Schachter *et al*., 2008; Griffin *et al*., 2014; Zilberberg *et al*., 2015). Recently Haas *et al.* (2020) conducted a prospective double-blinded randomized controlled trial comparing the number of oocytes, mature oocytes, blastocysts, top-quality blastocysts, implantation rate, ongoing pregnancy rate and live birth rate in patients with dual trigger or hCG alone trigger. They showed that using the dual trigger for final follicular maturation increases the number of oocytes, mature oocytes and number of blastocysts (total and top-quality) compared to triggering with hCG alone (Haas *et al*., 2020).

Some investigators evaluated the effect of dual trigger in poor responder patients. Oliveira *et al*. (2016) showed that this protocol increased the number of oocytes retrieved, mature oocytes and embryo fertilized when compared to patients who were treated with hCG alone trigger (Oliveira *et al*., 2016). Similar results were also found by Maged *et al*. (2021). Zhang *et al*. (2017) studied whether a dual trigger could improve the IVF outcomes of patients with poor ovarian response. They evaluated 1350 patients undergoing IFF cycles and randomly assigned in four experimental groups (Group A, 5000 IU hCG, n=328; Group B, 5000 IU hCG plus 0.1 mg GnRHa, n=386; Group C, 10,000 IU hCG, n=363; Group D, 10,000 IU HCG plus 0.1 mg GnRHa, n=312). Group A was compared with group B and group C was compared with group D. They showed that dual trigger groups showed significantly higher number of oocytes collected and number of mature oocytes compared with their respective HCG trigger group (*p*<0.001). Oocyte retrieval rate and percentage of mature oocytes retrieved were also both significantly higher in the dual trigger groups (*p*<0.001) (Zhang *et al*., 2017).

Another group conducted a retrospective cohort to evaluate patients with a fertilization rate lower than 20% in at least two prior ICSI cycles that subsequently underwent another ICSI cycle with hCG trigger alone or with dual. They observed that dual trigger improved ICSI outcomes in patients with a history of poor fertilization after standard hCG trigger alone (Elias *et al*., 2017). Recently, similar results about dual triggering oocyte final maturation in patients with a previous r-hCG triggered ICSI cycle were found. They demonstrated that dual trigger regimen improved response to COS, and showed better laboratorial and clinical outcomes (Setti *et al*., 2022).

Recently, Kim *et al*. (2020) evaluated the effect of dual trigger on oocyte maturation in young women with decreased ovarian reserve. They showed that a dual trigger was more beneficial than hCG alone in terms of mature oocyte cryopreservation for young women with DOR (Kim *et al*., 2020). A retrospective analysis by Lin *et al*. (2019) also showed that for patients with DOR, dual trigger can significantly improve the live birth rate, clinical pregnancy rate, and fertilization rate (Lin *et al*., 2019).

According to the studies mentioned above it is possible to state that the use of GnRHa combined with hCG in inducing final oocyte maturation is an excellent alternative strategy, regardless of whether the patient is a good or poor responder or with immature oocytes. The results presented in this article reinforce the evidence of improved outcomes of human reproduction treatments using dual triggering method. However it is important to mention that despite the dual trigger improve oocyte production, both in terms of number and maturity, this protocol for final oocyte maturation has already been shown to be associated with a significantly increased risk of severe OHSS compared to GnRH alone (O’Neill *et al*., 2016). Moreover, recently Li *et al*. (2022) showed that when dual trigger was compared with the hCG-only trigger group, similar embryological and clinical outcomes were achieved, although more oocytes were retrieved in the dual trigger group. They believe that there may be no extra benefit from dual triggering, and that it should not be recommended for routine use in the general population undergoing progestin-primed ovarian stimulation protocols (Li *et al*., 2022).

Although the dual trigger protocol was superior in terms of increasing the total number of oocytes retrieved and the number of oocytes inseminated compared to the hCG protocol, the results of the present study showed that regarding to the number of follicles, the number of MII oocytes recovered, number of fertilized oocytes and number of IVM, both protocols were equivalent. Similar results were also found by other authors (Ding *et al*., 2017; Eser *et al.*, 2018; Ben-Haroush *et al.*, 2020; Oliveira *et al*., 2021; Shapiro *et al*., 2021; Dong *et al*., 2022; Zhou *et al*., 2022).

Eser *et al*. (2018) observed no clinical difference when a dual trigger was used instead of an hCG trigger in poor responder women. They believe that aneuploidy and poor oocyte maturity could be the reason why dual triggering did not show benefits in poor responder patients (Liu, 2016; Eser *et al*., 2018). Ben-Haroush *et al*. (2020) showed that for those patients with low oocyte maturation rate in previous cycle triggered with hCG alone, the dual trigger significantly increased oocyte maturation rate. However, co-administration of GnRHa and hCG did not seemed to be beneficial for unselected population of patients. Their study also showed that the fertilization rate was similar between dual trigger and hCG protocol (mean±SD: 58±24 and 53±35, respectively; *p*=0.120) (Ben-Haroush *et al*., 2020). Similar results were shown recently by Oliveira *et al*. (2021). They evaluated prospectively 114 patients (unselected population) undergoing IVF cycles with dual triggering versus hCG alone. As shown here, the number of oocytes MII retrieved was not significantly different between hCG and dual trigger groups (mean±SD: 7.2±6.1 and 7.5±5.4, respectively; *p*=0.5022) (Oliveira *et al*., 2021).

Considering the risk of OHSS as a side effect of hCG, Shapiro *et al.* (2021) evaluated whether a low dose hCG (1000u) dual trigger would provide adequate luteal phase support to sustain a successful pregnancy without increasing OHSS rates compared with an hCG only trigger. They showed that dual trigger group patients had more oocyte MII retrieved (mean±SD: 13.5±6.6 and 11.5±6.1, respectively) and more fertilized oocyte rate (mean±SD: 0.80±0.20 and 0.77±0.23, respectively). Although women in the dual trigger group had a better prognosis based on age and AMH level and better stimulation outcomes, pregnancy outcomes were significantly lower which means that 1000u hCG dose was not enough to provide adequate luteal phase support. Of note, the dual trigger group had younger patients than the hCG group (33.6 and 34.1 year, respectively) and the AMH concentration was also higher in patients from dual trigger group when compared with hCG group (mean ± SD: 6.3±4.5 and 4.9±3.8 ng/mL, respectively) (Shapiro *et al*., 2021). In our study both groups were not heterogeneous for patients age as seen above. This may explain, at least in part, the similarity between groups considering those IVF outcomes.

Some studies have shown that the results of ARTs are similar between the double-triggered and hCG-only protocols. In a systematic review conducted by Ding *et al*. (2017) which included four eligible studies and involved 527 patients it was shown that the number of oocytes MII retrieved (*p*=0.62), number of fertilized oocytes (*p*=0.50), good-quality embryos (*p*=0.77) and implantation rate had no significant differences between the two groups (Ding *et al*., 2017). In the present study the implantation rate was not evaluated, but a possible explanation for an improved implantation rate when using dual trigger is that GnRH may increase endometrial receptivity. There is evidence of GnRH receptors (GnRHr) in human endometrium (Maggi *et al*., 2016). Since GnRH-a have a stronger binding affinity to the GnRHr than the GnRH antagonists it removes the GnRH antagonist from the GnRHr in the endometrium and activates the GnRHr (Schachter *et al*., 2008; Kim *et al*., 2014).

Recent studies confirmed the similarity between both protocols for triggering final oocyte maturation. Dong *et al*. (2022) investigated retrospectively whether a dual trigger for final oocyte maturation with a combination of a single dose of GnRH agonist and a standard dose of hCG could improve the reproductive outcomes compared with conventional hCG trigger alone. They showed that there were no differences between dual trigger and hCG protocols regarding fertilization rate (24.6% and 29.8%, respectively; *p*=0.528), number of follicles on trigger day (mean±SD: 6.86±3.55 and 6.4±3.25, respectively; *p*=0.476) among other characteristics of ovarian stimulation and laboratory indicators. However, they suggested that dual trigger protocol may cause a higher rate of miscarriage, but other clinical trials with a large population must be done to confirm this finding (Dong *et al*., 2022). In the same year, Zhou *et al*. (2022) conducted an open-label randomized controlled trial to investigate whether a dual trigger with a combination of GnRHa and low-dose hCG is superior to single hCG and/or single GnRHa trigger in advanced-age women undergoing IVF/ICSI treatment. They observed that there were no differences among dual trigger and hCG-alone protocols regarding the number of follicles (mean±SD: 4.64±2.80 and 4.29±2.84, respectively; *p*=0.190), number of matured oocytes (mean±SD: 3.54±2.51 and 2.78±2.10, respectively; *p*=0.061), fertilization rate at ICSI (82.1% and 83.3, respectively; *p*= .711) and fertilization rate at IVF (76.6% and 81.1%, respectively; *p*= .096) (Zhou *et al*., 2022). Thus, the efficacy of dual triggering for final oocyte maturation in the GnRH antagonist cycle remains unclear and has not been thoroughly investigated.

## CONCLUSION

Dual trigger protocol increased the total number of oocytes retrieved and the number of oocytes inseminated compared to the hCG alone. On the other regarding to the number of follicles, the number of MII oocytes recovered, number of fertilized oocytes and number of IVM, both protocols were equivalent.

However, larger prospective randomized controlled studies are needed to evaluate whether a dual trigger improves human embryo *in vitro* production and ICSI outcomes.
